# Inhibition of WEE1 Is Effective in *TP53*- and *RAS*-Mutant Metastatic Colorectal Cancer: A Randomized Trial (FOCUS4-C) Comparing Adavosertib (AZD1775) With Active Monitoring

**DOI:** 10.1200/JCO.21.01435

**Published:** 2021-09-18

**Authors:** Jenny F. Seligmann, David J. Fisher, Louise C. Brown, Richard A. Adams, Janet Graham, Philip Quirke, Susan D. Richman, Rachel Butler, Enric Domingo, Andrew Blake, Emma Yates, Michael Braun, Fiona Collinson, Rob Jones, Ewan Brown, Emma de Winton, Timothy C. Humphrey, Mahesh Parmar, Richard Kaplan, Richard H. Wilson, Matthew Seymour, Timothy S. Maughan

**Affiliations:** ^1^ Leeds Institute of Medical Research, University of Leeds, Leeds, United Kingdom; ^2^ MRC Clinical Trials Unit at UCL, London, United Kingdom; ^3^ Centre for Trials Research University and Velindre NHS Trust, Cardiff, United Kingdom; ^4^ Beatson Hospital, Glasgow, United Kingdom; ^5^ Bristol Genetics Laboratory, Bristol, United Kingdom; ^6^ MRC Oxford Institute for Radiation Oncology, University of Oxford, Oxford, United Kingdom; ^7^ Christie Hospital, Manchester, United Kingdom; ^8^ School of Medicine, Cardiff University, Cardiff, United Kingdom; ^9^ Western General Hospital, Edinburgh, United Kingdom; ^10^ Royal United Hospital, Bath, United Kingdom

## Abstract

Outcomes in *RAS*-mutant metastatic colorectal cancer (mCRC) remain poor and patients have limited therapeutic options. Adavosertib is the first small-molecule inhibitor of WEE1 kinase. We hypothesized that aberrations in DNA replication seen in mCRC with both *RAS* and *TP53* mutations would sensitize tumors to WEE1 inhibition.

**METHODS:**

Patients with newly diagnosed mCRC were registered into FOCUS4 and tested for *TP53* and *RAS* mutations. Those with both mutations who were stable or responding after 16 weeks of chemotherapy were randomly assigned 2:1 between adavosertib and active monitoring (AM). Adavosertib (250 mg or 300 mg) was taken orally once on days 1-5 and days 8-12 of a 3-week cycle. The primary outcome was progression-free survival (PFS), with a target hazard ratio (HR) of 0.5 and 80% power with a one-sided 0.025 significance level.

**RESULTS:**

FOCUS4-C was conducted between April 2017 and Mar 2020 during which time 718 patients were registered; 247 (34%) were *RAS/TP53*-mutant. Sixty-nine patients were randomly assigned from 25 UK hospitals (adavosertib = 44; AM = 25). Adavosertib was associated with a PFS improvement over AM (median 3.61 *v* 1.87 months; HR = 0.35; 95% CI, 0.18 to 0.68; *P* = .0022). Overall survival (OS) was not improved with adavosertib versus AM (median 14.0 *v* 12.8 months; HR = 0.92; 95% CI, 0.44 to 1.94; *P* = .93). In prespecified subgroup analysis, adavosertib activity was greater in left-sided tumors (HR = 0.24; 95% CI, 0.11 to 0.51), versus right-sided (HR = 1.02; 95% CI, 0.41 to 2.56; interaction *P* = .043). Adavosertib was well-tolerated; grade 3 toxicities were diarrhea (9%), nausea (5%), and neutropenia (7%).

**CONCLUSION:**

In this phase II randomized trial, adavosertib improved PFS compared with AM and demonstrates potential as a well-tolerated therapy for *RAS/TP53*-mutant mCRC. Further testing is required in this sizable population of unmet need.

## INTRODUCTION

Targeting the cellular DNA damage response (DDR) has been an effective therapeutic strategy in several tumor sites, including ovarian and pancreatic cancer.^1,2^ These agents can be used as monotherapy in cancers with defective DDR, where we might anticipate a synthetic lethality interaction: two pathways together perform an essential function, and the loss of one pathway (eg, because of mutation) is tolerated but the loss of both pathways leads to cell death.^3^

**CONTEXT**

**Key Objective**
To test if adavosertib, which is a small-molecule inhibitor of the WEE1 kinase, is effective as monotherapy in patients with *RAS/TP53*-mutant metastatic colorectal cancer (mCRC) as maintenance therapy following induction chemotherapy.
**Knowledge Generated**
In this phase II randomized trial, adavosertib was well-tolerated and improved progression-free survival in *RAS/TP53*-mutant mCRC compared with active monitoring. Treatment effect may be affected by primary tumor location and *KRAS* subtype, with greater benefit seen in left-sided cancers and those with *KRAS* codon 12/13 mutations. *RAS/TP53* subgroup is a distinct moderately poor prognostic population.
**Relevance**
Adavosertib is a promising therapeutic agent in patients with *RAS/P53*-mutant mCRC, a poor prognostic population of unmet need, and was well-tolerated. This study demonstrates the potential of targeting the DNA damage response pathway in mCRC, which should be a research priority. Future studies of adavosertib should stratify patient outcomes according to primary tumor location and *RAS* subtype.

WEE1 is a nuclear tyrosine kinase that has a central role in cell cycle regulation, including being the key regulator of the G2/M checkpoint through actions on CDK1,^4^ optimizing DNA-histone stoichiometry before mitotic entry^4^ and modulation of CDK1/2 during the intra-S phase to block replication initiation.^5^ Inhibition of WEE1 causes unscheduled entry into mitosis, aberrant firing of replication origins leading to dNTP (Dithiobis [5-nitropyridine]) shortage and replication stress,^4^ and accumulation of DNA damage during S phase, leading to increased reliance on the G1/S checkpoint.^4^ Adavosertib (AZD1775) is the first small-molecule inhibitor of WEE1 kinase and has been tested in combination with chemotherapy and radiotherapy^6,7^ but more recently as monotherapy to generate synthetic lethality in tumors with DDR defects.^6^

There has been limited investigation into agents targeting the DDR in metastatic colorectal cancer (mCRC), mainly because of the lack of systematic identification of alterations in DDR genes.^8^ Here, we test adavosertib in *RAS*- and *TP53*-mutant (*RAS/TP53*-mut) mCRC, which we hypothesize would be sensitive to WEE1 inhibition. *TP53* is a key regulator of the G1/S checkpoint^9^; loss of function leads to dependence on the intra-S and G2/M checkpoints to detect DNA damage and initiate repair.^10^ In preclinical studies, AZD1775 possessed preferential killing effect in *TP53*-deficient compared with *TP53* wild-type tumors.^11^ Mutant *RAS,* as well as recognized actions through downstream mitogen-activated protein kinase B (MAPK-AKT) pathway signaling, also drives cell cycle progression leading to replication stress during S phase.^12^ In preclinical studies, mutant *RAS* drives cells into S phase through regulation of the CDK4 or CDK6 complex and provides sustained mitogenic signals through sustained CDK2 activity. These effects activate the replication stress response including checkpoint activation.^13^ Theoretically, *RAS/TP53*-mut tumors will be highly vulnerable to adavosertib, with G1 checkpoint failure, evidence of replication stress, and reliance on the intra-S phase and G2/M checkpoints.

The FOCUS4 trial program was an adaptive molecularly stratified umbrella platform trial that evaluated the safety and efficacy of novel treatments in targeted biomarker subgroups within a phase II/III trial setting in the interval after 16 weeks of first-line therapy of mCRC. The design has been published separately,^14^ and the trial schema, registration, and biomarker methods are provided in the Data Supplement (online only). Here, we report the findings of FOCUS4-C, which tested the safety and efficacy of adavosertib in patients with *RAS/TP53*-mut mCRC compared with active monitoring (AM) and has achieved disease stability following induction chemotherapy.

## METHODS

### Trial Approvals, Patient Eligibility, and Recruitment

The trial and subsequent amendments were approved by the UK National Ethics Committee Oxford—Panel C (reference 13/SC/0111) and by the relevant regulatory body MHRA (CTA No. 20363/0400/001 and EudraCT No. 2012-005111-12).

**FIG 1.**
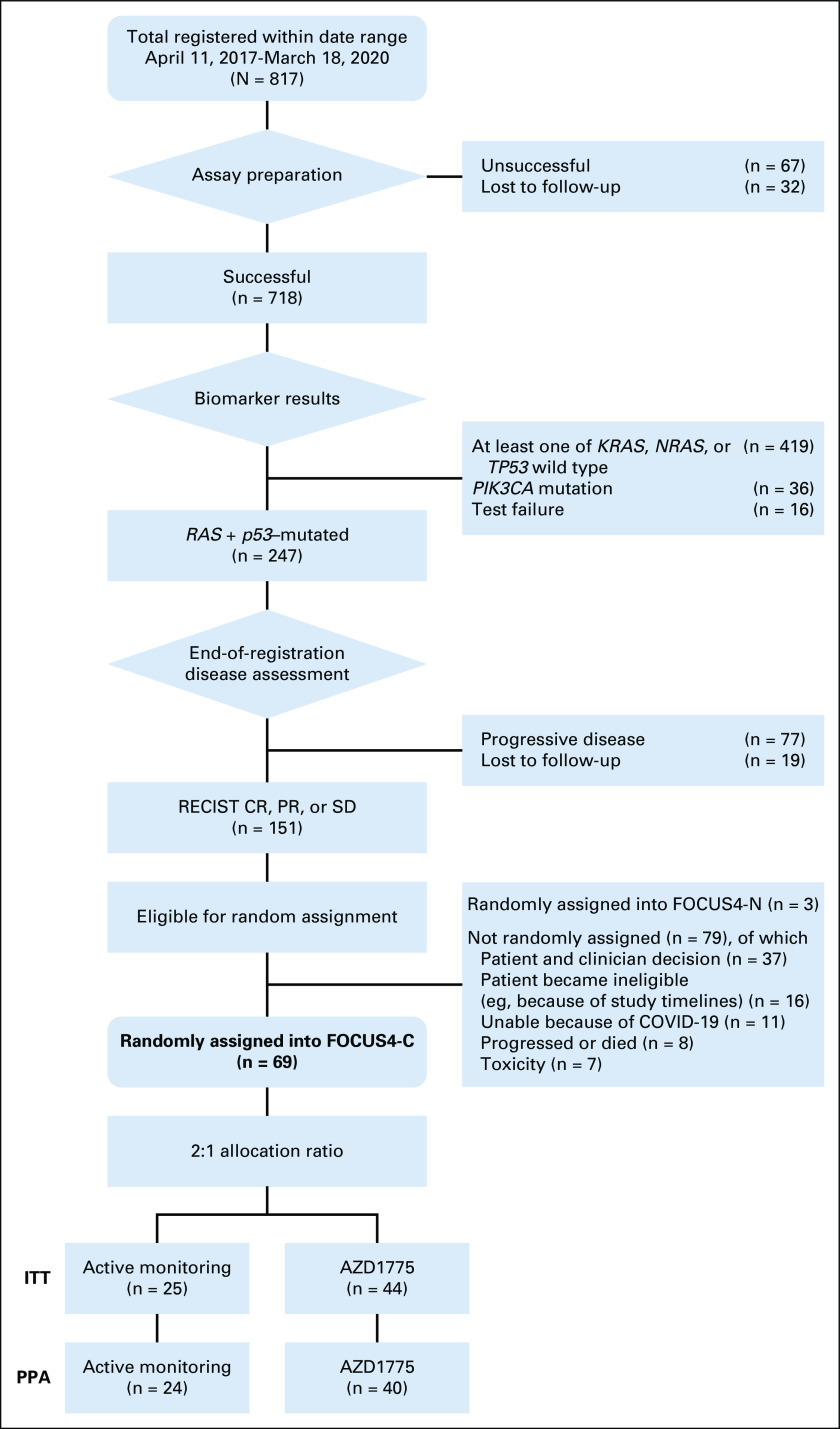
Flowchart of patients through the trial. CR, complete response; ITT, intention-to-treat; PPA, per-protocol analysis; PR, partial response; SD, stable disease.

Patients age more than 18 years with newly diagnosed mCRC were registered into the FOCUS4 trial program, while undergoing induction chemotherapy, from a total of 88 UK hospitals. Following registration, a tumor sample was tested using next generation sequencing platform for stratification into molecular subtypes including *BRAF*, *PIK3CA*, *TP53*, and *RAS* mutations (Fig 1 and Data Supplement). Patients were required to provide written informed consent for both tissue testing and entry into any of the randomized sub-trials including FOCUS4-C.

Patients were randomly assigned into the FOCUS4-C trial in a subset of 25 hospitals between July 2017 and March 2020. Patients were eligible if their tumor had both *RAS* and *TP53* mutations and they had disease stability or response as assessed by computed tomography (CT) scan at the end of 16 weeks of induction chemotherapy, at which point the chemotherapy ceased and the patient was randomly assigned. Patients required a baseline CT scan 4 weeks before random assignment, a minimum 3-week washout period between the last dose of chemotherapy or biologic therapy and the first dose of adavosertib, adequate renal (creatinine clearance > 50 mL/min) and liver function, a WHO performance status of 0-1, and no evidence of prolonged QT interval on ECG.

### Trial Procedures

Adavosertib was supplied by AstraZeneca Ltd (Cambridge, UK); packaging, labeling, and distribution were undertaken by Fisher Services (Horsham, UK). Patients randomly assigned to adavosertib continued the drug until disease progression, death, or intolerable toxicity. The first 21 patients received adavosertib 250 mg once daily, on days 1-5 and 8-12 of a 3-week cycle. The next 23 patients received adavosertib 300 mg once daily, on the same schedule. Patients took an oral 5HT3 antagonist with each dose, and oral dexamethasone 4 mg was given on day 1 and day 8 of each cycle unless clinically contraindicated.

Because of the mandatory supportive medication for nausea and vomiting for which a placebo was not available, blinding was not possible, and AM was used as the control arm. Patients randomly assigned to AM followed the same follow-up schedule and remained off any other anticancer treatment until clinical or radiologic evidence of disease progression.

Patient tumor status was assessed at the treating hospital every 8 weeks by CT scan, according to RECIST, version 1.1.^15^ Toxicities and symptoms were assessed locally every 4 weeks, using the National Cancer Institute's Common Terminology Criteria for Adverse Events (version 3.0). Patients remained on trial until disease progression occurred, at which point the patient was recommended to restart the same chemotherapy that was used in the induction phase. Treatment was stopped in the event of grade 3 or worse toxic effects or persistent toxicities judged medically significant or not tolerated by the patient, until the toxicity resolved to grade 1 or better.

### Statistical Methods

A full description of the statistical methods is provided in the Data Supplement. In summary, patients were allocated to either adavosertib or AM, using a 2:1 allocation ratio by minimization with a 20% random element. All analyses were performed according to a predefined statistical analysis plan using Stata (version 16.1; Stata Corporation, TX). The primary outcome measure was progression-free survival (PFS), and the prespecified primary efficacy analysis was a per-protocol analysis (PPA) using Cox regression adjusting for minimization factors. Intention-to-treat (ITT) and unadjusted models were also performed as secondary analyses. Sample size calculations were based upon a target hazard ratio (HR) of 0.5 with 80% power and .025 one-sided alpha requiring a target of 26 PFS events in the control arm for final analysis.

## RESULTS

### Recruitment and Patient Characteristics

The FOCUS4 trial program ran between January 2014 and March 2020. FOCUS4-C ran between April 2017 and March 2020, during which time 817 patients were registered, of whom 718 underwent successful biomarker profiling (Fig 1 and Data Supplement). Two hundred forty-seven patients (34%) had tumors confirmed with both *RAS* and *TP53* mutations (*RAS/TP53*-mut). Of these, 151 had stable or responding disease after 16 weeks of first-line treatment and 69 were randomly assigned using a 2:1 ratio: 44 to adavosertib and 25 to AM. Of the remaining eligible 82, two chose to be randomly assigned into the concurrent FOCUS4-N trial and others chose not to be randomly assigned into FOCUS4 for reasons such as toxicity from first-line therapy or patient-clinician choice to seek alternative pathways.

Table 1 summarizes the patient baseline characteristics. There were some minor imbalances, which are corrected for in the adjusted analysis (primary model). There were no differences in the frequency of other molecular alterations between the groups. There were no significant differences between the registration period chemotherapy regimens in the adavosertib and AM arms.

**TABLE 1.**
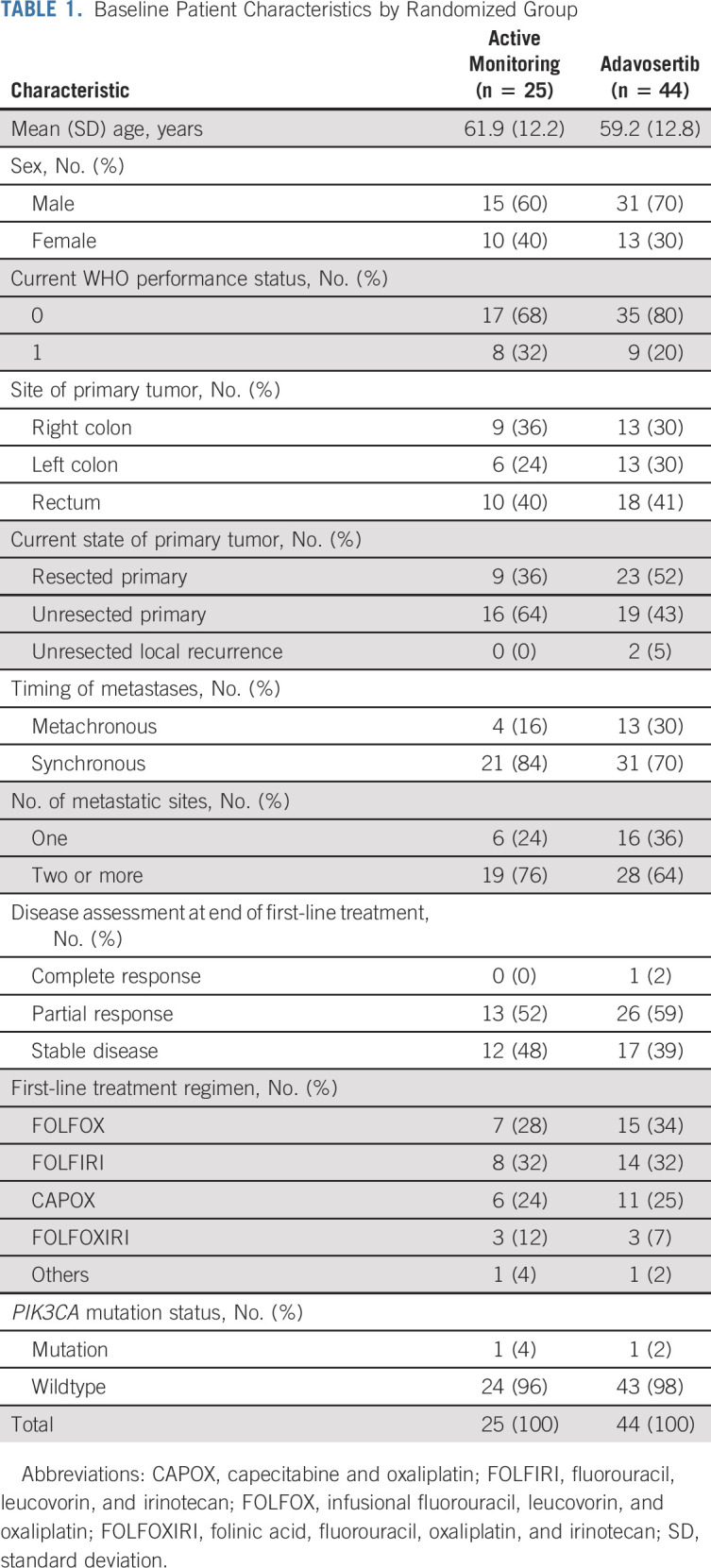
Baseline Patient Characteristics by Randomized Group

### Primary Analysis: PFS (per-protocol)

Five patients were excluded from the PPA: four did not start treatment (adavosertib arm) and one was subsequently found to have had progressive disease at the point of random assignment (AM arm). One patient was censored early when they received fluorouracil as anticancer treatment before progression (AM arm).

**FIG 2.**
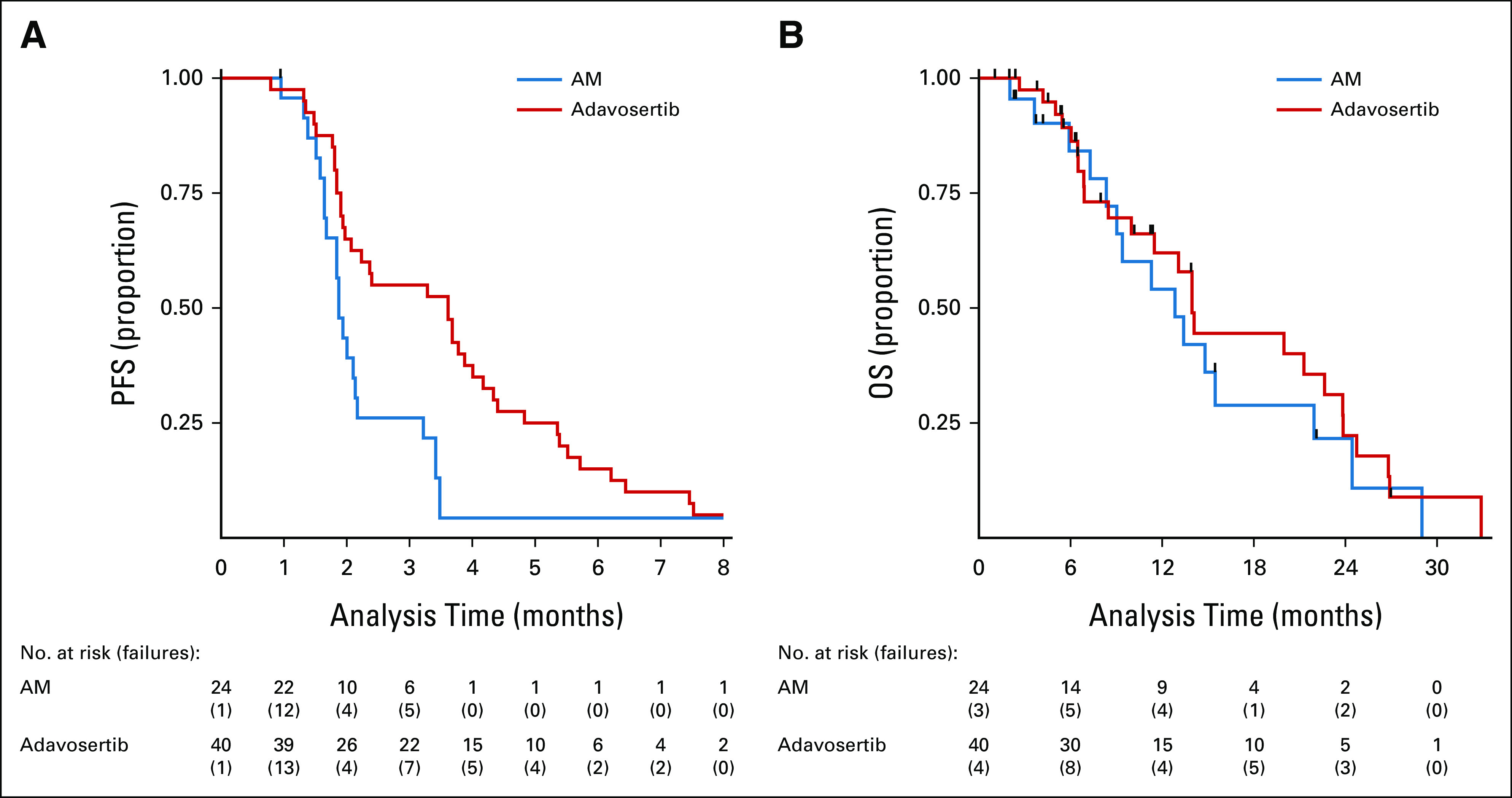
(A) PFS (primary analysis) in PPA population: Cox regression, adjusted for minimization factors—HR = 0.35 (95% CI, 0.18 to 0.68), *P* = .0022. Minimization factors: location of primary tumor (left, right, and rectum), baseline WHO performance status, baseline disease assessment, number of metastases, and first-line therapy (fluoropyrimidine, oxaliplatin or irinotecan, and monoclonal antibody). (B) OS (secondary analysis) in PPA population: Cox regression, adjusted for minimization factors—HR = 0.86 (95% CI, 0.39 to 1.86), *P* = .70. Minimization factors: location of primary tumor (left, right, and rectum), baseline WHO performance status, baseline disease assessment, number of metastases, and first-line therapy (fluoropyrimidine, oxaliplatin or irinotecan, and monoclonal antibody). AM, active monitoring; HR, hazard ratio; OS, overall survival; PFS, progression-free survival; PPA, per-protocol analysis.

Within the primary PPA (n = 64), there were 40 of 40 PFS events in the adavosertib arm and 22 of 24 in the AM arm. Patients treated with adavosertib had a longer PFS than those on AM (3.61 *v* 1.87 months). Both unadjusted HR (0.52; 95% CI, 0.30 to 0.89; *P* = .022) and adjusted HR (0.35; 95% CI, 0.18 to 0.68; *P* = .0022) were statistically significant. Kaplan-Meier curves are provided in Figure 2.

### PFS (ITT)

All patients were included in the ITT analysis, but four patients were censored the day after random assignment: three in the adavosertib arm (two because of patient withdrawal and one without any post–random assignment CT scan assessments) and one in the AM arm without any post–random assignment CT scan assessments.

There were 41 of 44 PFS events in the adavosertib arm and 23 of 25 in the AM arm. Consistent with the PPA, the ITT PFS analysis shows a PFS advantage with adavosertib over AM in both the unadjusted (HR = 0.55; 95% CI, 0.32 to 0.94; *P* = .032) and adjusted analyses (HR = 0.40; 95% CI, 0.21 to 0.75; *P* = .0051).

### Overall Survival (ITT)

There were 27 of 44 deaths in the adavosertib arm and 16 of 25 in the AM arm. There was no significant overall survival (OS) benefit with adavosertib compared with AM (median survival 14.0 *v* 12.8 months; unadjusted HR = 0.79; 95% CI, 0.42 to 1.48, *P* = .47; adjusted HR = 0.92; 95% CI, 0.44 to 1.94, *P* = .93; Fig 2).

### Tumor Control

Adavosertib was associated with a higher proportion of patients with disease control compared with AM (47% *v* 28% at any time during the trial), including one patient with a documented partial response to adavosertib (Data Supplement).

### Subgroup Analyses

**FIG 3.**
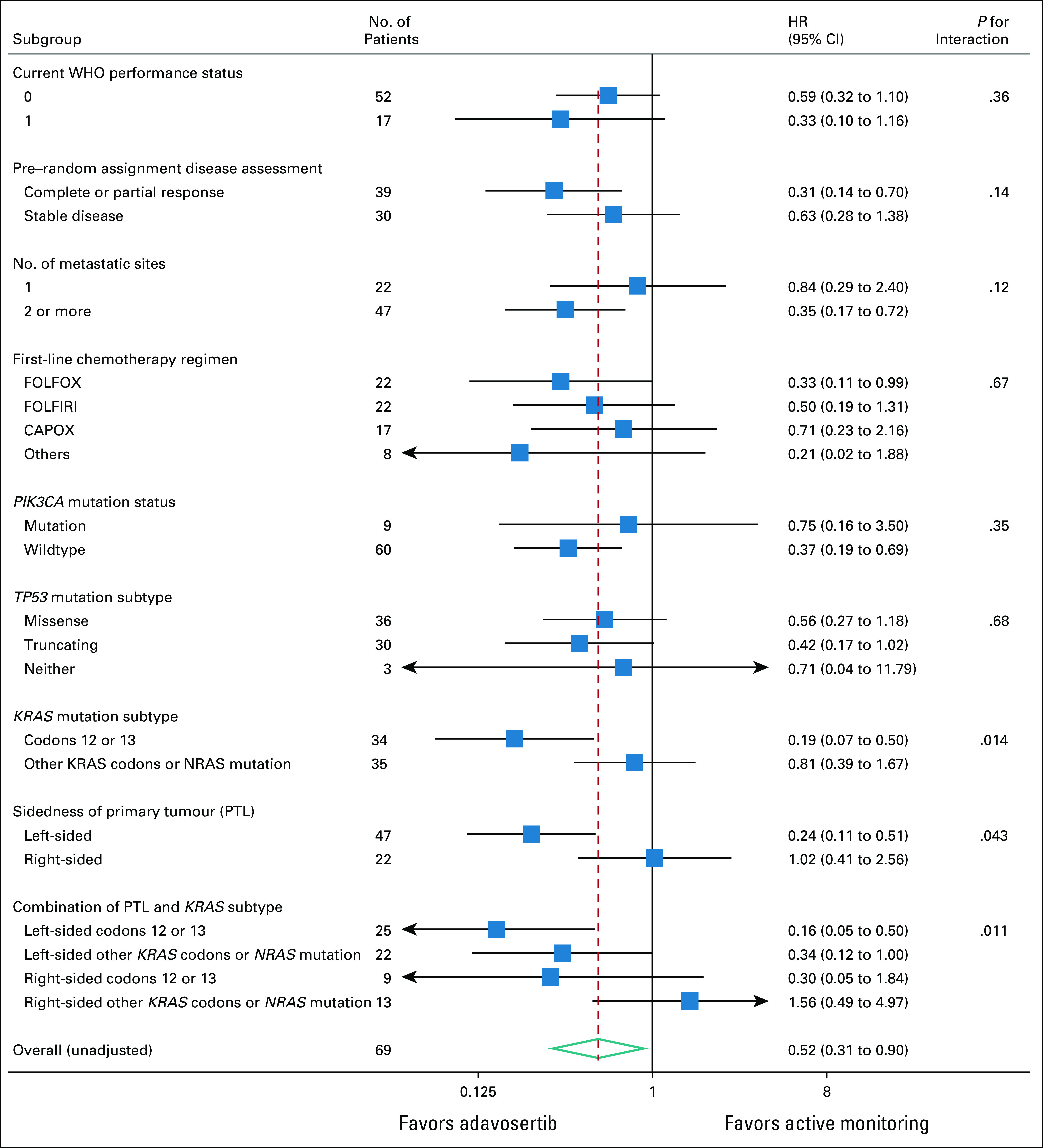
Subgroup analyses for PFS by intention to treat. CAPOX, capecitabine and oxaliplatin; FOLFIRI, fluorouracil, leucovorin, and irinotecan; FOLFOX, infusional fluorouracil, leucovorin, and oxaliplatin; PFS, progression-free survival; PTL, primary tumor location.

The impact of adavosertib versus AM on PFS was explored in prespecified subgroups (Fig 3). The most marked difference in effect was for primary tumor location (PTL): patients with a right PTL had no PFS advantage with adavosertib compared with AM (1.87 *v* 1.91 months; HR = 1.02; 95% CI, 0.41 to 2.56), whereas those with a left PTL did (3.61 *v* 1.87 months, HR = 0.24; 95% CI, 0.11 to 0.51; interaction *P* = .043; Data Supplement).

This prompted an unplanned subgroup analysis of PTL on OS, and although the numbers of events were low, the interaction was even more marked (Data Supplement). Median OS was 14.1 versus 11.3 months for adavosertib versus AM in left PTL (adjusted HR = 0.37; 95% CI, 0.15 to 0.87) but was 6.5 versus 15.5 months in right PTL (HR = 6.5; 95% CI, 0.72 to 6.43; interaction *P* = .0032). In terms of response, 38% of right-sided adavosertib patients versus 42% of right-sided AM patients reported disease stability or response at least once while on trial, whereas for left-sided tumors, the figures were 53% versus 19%.

Patients who had responded to induction chemotherapy (*v* stable disease) and who had two or more metastatic sites appeared to benefit more from adavosertib, albeit to a lesser degree (interaction *P* value = .14 for response to induction; *P* = .12 for number of metastatic sites; Fig 3).

### External Analyses to Further Characterize the *RAS/TP53*-Mut Biomarker Population

**FIG 4.**
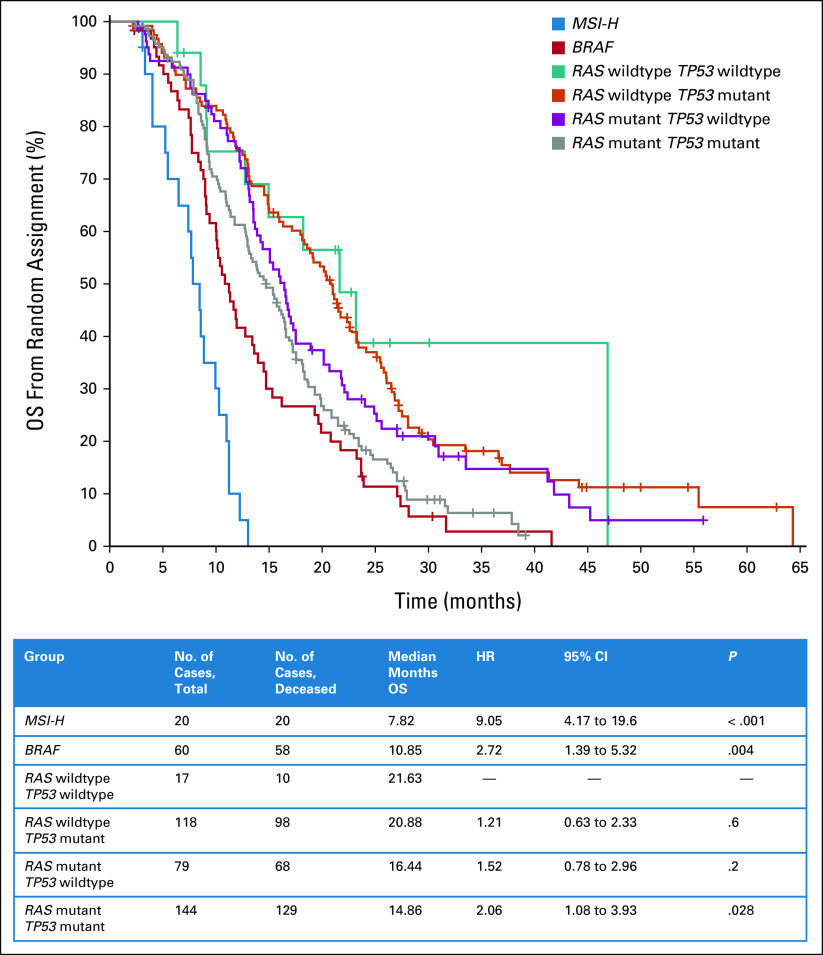
Prognostic impact of biomarker subgroups on OS in previous FOCUS trial. HR, hazard ratio; MSI-H, microsatellite instability-high; OS, overall survival.

The *RAS/TP53*-mutant population has not been previously described. To understand the prognostic implication of this alteration, we analyzed the outcomes of a subset (n = 438) of patients from the FOCUS trial in whom the S:CORT consortium had analyzed a wider panel of CRC genes including *KRAS*, *NRAS*, *BRAF*, *MSI*, and *TP53*. The *RAS/RAF* wild-type group was the reference population (median OS 21.6 months). The *RAS/TP53*-mutant population is distinct from either mutation alone (*RAS* or *TP53*) and had a worse prognosis than either in isolation with a median OS of 14.9 months (HR = 2.06; 95% CI, 1.08 to 3.93; *P* = .028; Fig 4). This suggests that the *RAS/TP53*-mut population is a poor-prognosis subgroup but not as marked as for patients with a *BRAF* mutation or microsatellite instability-high tumor.

These data are consistent with the finding that during the registration period of FOCUS4, 33% of patients in the *RAS/TP53*-mut population experienced progression during the first 16 weeks of chemotherapy. This is similar to the rate in the *BRAF*-mutant group (34% progressed) but higher than that seen in *RAS*-mutant (24%) and all wild-type (22%) subgroups (Data Supplement).

### Effect of *RAS* and *TP53* Mutation Subtypes on Adavosertib Activity

We observed that patients with *KRAS* codon 12/13 mutations had a significant benefit from adavosertib (*P* for interaction = .014; Fig 3), whereas no detectable benefit was observed in those with *KRAS* mutations at other codons or with *NRAS* mutation. Furthermore, the interaction effects of *KRAS* subtype and of PTL on PFS may be additive as there is a significant benefit from adavosertib within the subgroup of left PTL *KRAS* codon 12/13 subtypes (HR = 0.16; 95% CI, 0.05 to 0.50) and a clear disbenefit within the subgroup of right PTL noncodon 12/13 subtypes (HR = 1.56; 95% CI, 0.49 to 4.97; Data Supplement). The subtype of *TP53* mutation or the co-occurrence of *PIK3CA* mutation did not affect outcome.

### Toxicity and Compliance

**FIG 5.**
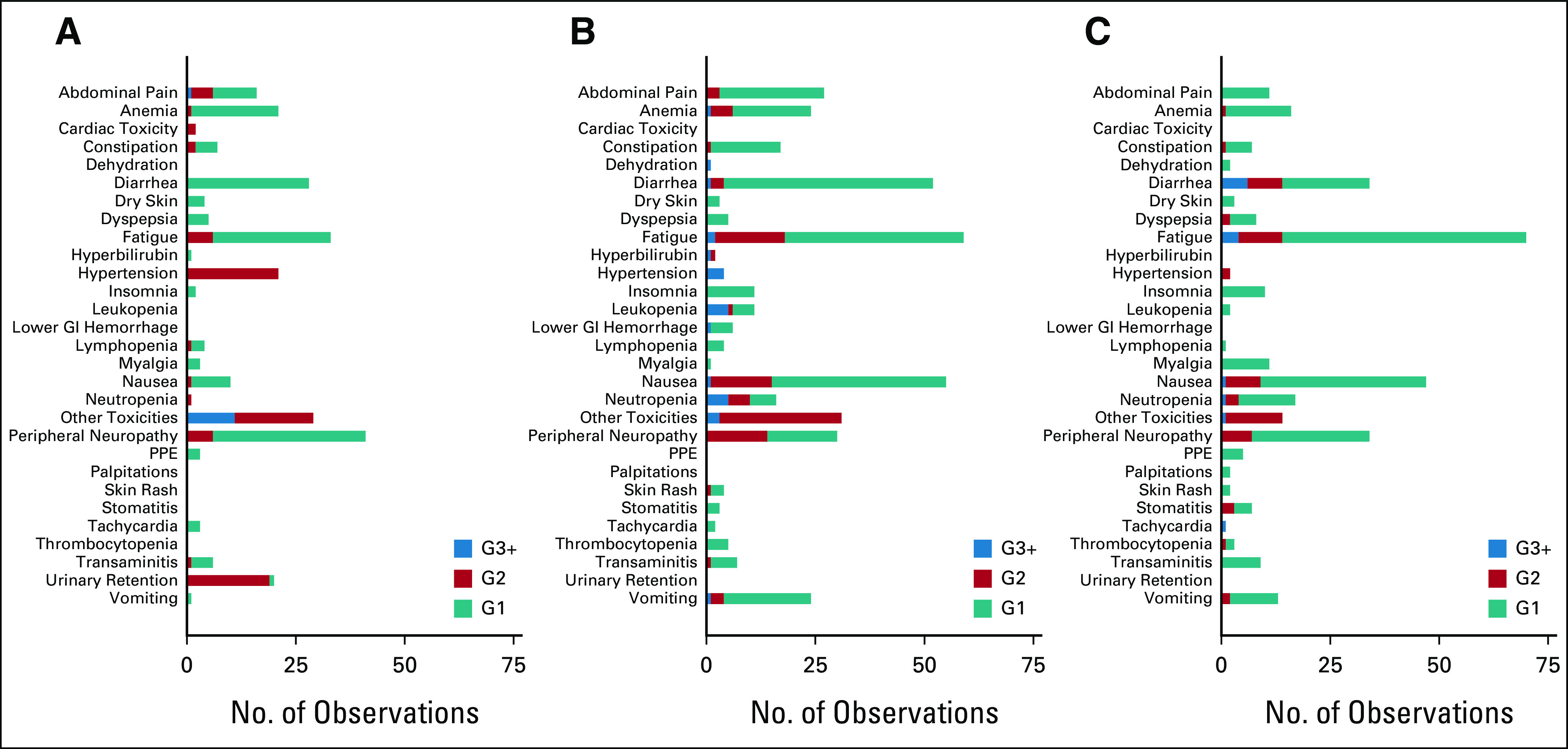
Cumulative reported toxicity, within FOCUS4-C treatment groups and with initial AZD1775 doses separated: (A) active monitoring (n = 25), (B) AZD1775 250 mg (n = 23), and (C) AZD1775 300 mg (n = 21). G, grade; PPE, palmar plantar erythema.

There was good compliance with randomized allocation, and adavosertib was generally well-tolerated (Fig 5 and Data Supplement). Compared with AM, adavosertib was associated with increased reported toxicity (≥ grade 1), most notably increased frequency of diarrhea (61% *v* 28%), fatigue (75% *v* 56%), nausea (68% *v* 32%), and vomiting (41% *v* 4%). However, the majority of such toxicity was of low grade, with 9% in the adavosertib arm reporting diarrhea of ≥ grade 3, 11% fatigue, 5% nausea, and 2% vomiting, versus none of each in the AM arm. As described, during the trial, there was an increase in the dose of adavosertib from 250 mg to 300 mg. The higher dose was associated with an increased frequency of grade 3 diarrhea (14% *v* 4%), but otherwise the toxicity profile was similar, and with similar rates of dose modifications and delays.

### Impact of Adavosertib Dosing

As described, during the trial, there was an increase in the dose of adavosertib from 250 mg to 300 mg. PFS was 2.2 months (HR = 0.58; 95% CI, 0.31 to 1.06) with the 250-mg dose and 3.7 months (HR = 0.47; 95% CI, 0.25 to 0.89) with the 300-mg dose; this difference was nonsignificant (*P* = .48; Data Supplement). Between the 250-mg and 300-mg doses, there was an increased frequency of grade 3 diarrhea (4% *v* 14%), but otherwise the toxicity profile was similar. There were similar rates of dose modifications between the 250-mg and 300-mg doses: dose delays (16% *v* 7%), dose reductions (4% *v* 5%), and dose omissions (19% *v* 17%). A swimmer plot integrating the effects of adavosertib dose, randomized group, and PTL on PFS is shown in the Data Supplement.

## DISCUSSION

Here, we have reported that FOCUS4-C met its primary end point; patients with *RAS/TP53*-mutant mCRC had PFS advantage with adavosertib compared with AM following induction chemotherapy. These results are particularly encouraging as *RAS/TP53*-mutant mCRC is a poor prognostic population with limited treatment options. Adavosertib was well-tolerated at both doses evaluated.

The overarching aim of the FOCUS4 trial program was to test novel agents efficiently with specified biomarker subgroups in mCRC with the multi-arm, multi-stage design allowing for an early signal of drug inactivity^14^; thus, any demonstrated efficacy would require further confirmatory study to lead to practice change. FOCUS4-C represents a success of this approach, efficiently demonstrating promising activity of adavosertib within patients with *RAS/P53*-mutant mCRC, and will directly influence research practice in mCRC.

The intermittent treatment strategy used in FOCUS4 follows the demonstration of no detriment in OS in the MRC COIN trial. This is now further substantiated by an individual participant data meta-analysis.^16^ Thus, AM is an accepted standard of care following a few months of first-line therapy. FOCUS4 was specifically designed to use this window following first-line induction chemotherapy to test novel agents in specified biomarker groups, before the evolution of multiple resistance mechanisms.^14^

A prespecified analysis demonstrated that adavosertib activity was limited to left colon and rectal PTL, with little activity observed in right PTL. Having observed the significant subgroup effects on PFS, we investigated possible impact on OS. It is provocative to see that in the left-sided tumors, OS was significantly improved with median OS from random assignment increasing from 11.3 months to 14.1 months (HR = 0.40; 95% CI, 0.17 to 0.97). There is also a possibility of adverse effect on outcome in patients with right PTL. However, the number of patients and events was limited and thus, any conclusions need to be cautious in relation to this observed effect on OS in both subgroups. Differences in CRC by PTL are well-documented, in terms of biology, prognosis, and treatment response,^17^ but the mechanisms for differences of treatment efficacy by PTL are not well-understood.

An exploratory analysis showed that adavosertib had the most PFS effect in patients with *KRAS* codons 12/13/*TP53*-mutant tumors, with lesser activity in those with extended *KRAS*, or *NRAS* mutations; functional differences between *RAS* isoforms are documented.^18^ Despite the small sample sizes in FOCUS4-C, the PTL and *RAS* subtypes subgroup analyses showed interactions significant at the 5% level.

Although these subgroup analyses provide provocative results, we lack a mechanistic explanation for these differences in adavosertib effect; ongoing translational work shall investigate this. We would recommend that further clinical development of adavosertib in the *RAS/TP53*-mut mCRC population should not be limited by PTL or *RAS* subtype but should include close monitoring of patients with right PTL and extended *RAS* mutations to ensure that neither futility nor detriment are observed.

Although the clinical implications of the *RAS/TP53* mutation in mCRC are not well-studied, each alteration is individually well-characterized. Here, we have shown that the double *RAS/TP53*-mutant subgroup carries a moderately poor prognosis (Fig 4) and appears to confer a worse prognosis than either mutation in isolation. This biomarker subgroup has thus shown distinct prognostic and therapeutic relevance and so merits further study in translational work, existing data sets, and ongoing therapeutic trials in mCRC.

Adavosertib has demonstrated an acceptable safety profile; the main toxicity was diarrhea. Efficacy was noted at both the 250-mg and 300-mg doses, with a suggestion of additional activity with the higher dose. We would therefore recommend the 300-mg dosing to progress to further clinical studies in fit patients. However, in the treatment-refractory setting, the 250-mg dose may be more tolerable.

There are limitations to this study. We considered, and would have preferred, a placebo-controlled design; however, at the time of launching FOCUS4-C, high rates of nausea and vomiting had been observed in other adavosertib trials and high-dose steroid antiemetics were considered necessary. For this reason, both clinicians and patient representatives considered a placebo design unfeasible. Given the favorable safety data for single-agent adavosertib in FOCUS4-C, placebo-controlled design could be considered in the future. It is possible therefore that the PFS effect observed was influenced by investigator and patient preference to restart first-line chemotherapy sooner in the AM arm. However, a marked difference in effect was observed between the right and left PTL groups treated with adavosertib, suggesting a lesser effect on the primary analysis because of this potential bias. Additionally, the PFS end point was not centrally reviewed, but assessed in individual sites by RECIST criteria. A further limitation is that by testing adavosertib in the maintenance setting and requiring stability following induction chemotherapy, we have excluded the *RAS/TP53* patients with the worse outcome. We therefore cannot generalize the effect of adavosertib within this entire biomarker group.

In conclusion, adavosertib (AZD1775) has demonstrated promising activity compared with AM in patients with *RAS/TP53*-mut mCRC. This treatment benefit may relate to PTL and *KRAS* subtype. Given this clear demonstration of efficacy in an RCT and acceptable toxicity profile, future clinical development of adavosertib is warranted particularly as it may represent a future treatment opportunity in this sizable population of unmet need.

## Figures and Tables

**Table:**
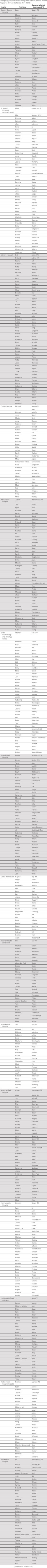
Participating Hospitals in Descending Order of the Number of Patients Registered With All Staff Listed (N = 2,076)

## Data Availability

Individual deidentified participant data (including data dictionaries) can be shared upon appropriate application to the MRC CTU at any time from full publication. Study protocols and statistical analysis plan have been provided in the Data Supplement with this manuscript. Going forward, it is proposed that data will be shared with an appropriate international collaborative repository to enable future IPD meta-analysis.

## APPENDIX 1. Full Authorship List for the FOCUS4 Trial Investigators

### Writing Committee for FOCUS4-C Trial

Seligmann J.F., Fisher D.J., Brown L.C., Adams R.A., Graham J., Quirke P., Richman S.D., Butler R., Domingo E., Blake A., Yates E., Braun M., Collinson F., Jones R., Brown E., De Winton E., Humphrey T.C., Parmar M., Kaplan R., Wilson R.H., Seymour M., and Maughan T.S.

### Trial Management Group

Maughan T.S. (Chair), Wilson R.H., Adams R.A., Seymour M., Seligmann J.F., Graham J., Wasan H., Pope M., Pope J., Samuel L., Shiu K., Church D., Middleton G., Steward W., Twelves C., Wellman S., Hodgkinson E., Stoner N., Beety J., Duggleby K., Dutton G., MRC CTU team (below), and laboratory staff (see below).

### MRC CTU Trial Coordinating Center

Brown L.C., Kaplan R., Parmar M., Fisher D.J., Campos M., Yates E., Santana S., Fiddament A., Harper L., Bara A., Pugh C., Bathia R., Letchemanan K., Przybył B., Bhogal S., Gopalakrishnan G., Purvis C., Diaz-Montana C., Mohamed F., Townsend S., Cragg W., Masters L., Van Looy N., and Rauchenberger M.

### Laboratories

Quirke P., Richman S.D., Hemmings G., Davis J., Gallop N., Wilkinson L., Butler R., Roberts H., Jasani B., White R., Dodds R., James M., and Morgan M.

### Independent Data-Monitoring Committee

Cameron D. (Chair), Souhami R., Peeters M., Billingham C., Griffiths G., and Brown J.

### Trial Steering Committee

Johnson P. (Chair), Rudd R., Whelan J., and Russell A.
